# A Novel Heterozygous Intronic Mutation in the* FBN1* Gene Contributes to* FBN1* RNA Missplicing Events in the Marfan Syndrome

**DOI:** 10.1155/2018/3536495

**Published:** 2018-05-29

**Authors:** Mario Torrado, Emilia Maneiro, Juan Pablo Trujillo-Quintero, Arturo Evangelista, Alexander T. Mikhailov, Lorenzo Monserrat

**Affiliations:** ^1^Institute of Health Sciences, University of A Coruña, A Coruña, Spain; ^2^Health in Code, A Coruña, Spain; ^3^Cardiology Department, Hospital General Universitari Vall d'Hebron, Barcelona, Spain

## Abstract

Marfan syndrome (MFS) is an autosomal dominantly inherited connective tissue disorder, mostly caused by mutations in the fibrillin-1 (*FBN1*) gene. We, by using targeted next-generation sequence analysis, identified a novel intronic* FBN1* mutation (the c.2678-15C>A variant) in a MFS patient with aortic dilatation. The computational predictions showed that the heterozygous c.2678-15C>A intronic variant might influence the splicing process by differentially affecting canonical versus cryptic splice site utilization within intron 22 of the* FBN1* gene. RT-PCR and Western blot analyses, using* FBN1* minigenes transfected into HeLa and COS-7 cells, revealed that the c.2678-15C>A variant disrupts normal splicing of intron 22 leading to aberrant 13-nt intron 22 inclusion, frameshift, and premature termination codon. Collectively, the results strongly suggest that the c.2678-15C>A variant could lead to haploinsufficiency of the FBN1 functional protein and structural connective tissue fragility in MFS complicated by aorta dilation, a finding that further expands on the genetic basis of aortic pathology.

## 1. Introduction

Aberrant pre-mRNA splicing is a frequent cause of human genetic disease and, according to some estimates, mutations affecting splicing comprise up to 60% of all disease-causing mutations in human genes [1, 2]. Molecular diagnostics, particularly with implementation of next-generation sequencing (NGS) platforms, continues to identify new causative mutations for rare genetic disorders such as Marfan syndrome (MFS) that is often crucial for appropriate patient management as well as for genetic risk assessment in families.

MFS is a heritable connective tissue disorder with clinical pathological manifestations in the skeletal, ocular, cardiovascular, and other systems [3]. In MFS, the development of structural cardiovascular alterations such as aortic aneurysms, mitral valve prolapse, dilated cardiomyopathy, and arrhythmia greatly contributes to patient morbidity and early mortality [4]. In situations of clinical uncertainty or presymptomatic counseling [5], molecular genetic testing and outcome predictions are an integral part of the diagnostic decision process [6–8].

MFS is often associated with and caused by numerous mutations (1847 currently reported, www.umd.be/FBN1/) in the fibrillin-1 (*FBN1*) gene [9, 10]. A fast-track* FBN1* mutation screening has therefore become a logical approach to add meaning to the clinical MFS diagnosis, recognition of at-risk cardiovascular MFS patients, and family planning counseling [11]. In fact, diagnostic screening identified a wide spectrum of pathogenic* FBN1 *mutations in MFS, resulting in* FBN1 *haploinsufficiency [8, 12]. However, for a substantial proportion of patients with MFS clinical phenotypes, DNA sequencing restricted to* FBN1* exons failed to identify putatively pathogenic mutations. In some of these situations, qualitative analysis of the* FBN1* mRNA in dermal fibroblasts from MFS patients revealed aberrant* FBN1 *pseudoexon inclusions due to intronic mutations which activate cryptic splice sites [13–17].

We describe a patient with a diagnosis of MFS where there is no mutation identified in the* FBN1* coding sequence. Targeted NGS of the genomic DNA from this patient revealed a novel variant located in intron 22 of the* FBN1 *gene. The results of both in silico and functional studies using minigene splicing assays strongly suggest that this variant leads to a cryptic acceptor splice site activation and subsequent frame shift and premature termination codon (PTC) in exon 23.

## 2. Materials and Methods

### 2.1. Patient Information

The index patient was a 32-year-old male with MFS. Clinical findings included aortic root dilatation (41 mm aortic sinus, 44 mm or 2.17cm/m2 ascending aorta), ectopia lentis, and typical skeletal manifestations. His father had aortic dilatation and died in his forties due to endocarditis after the implantation of an aortic prosthesis. His mother, his brother, and one paternal aunt were clinically unaffected. Since no relevant mutation was identified in a previous genetic study limited to exons and close flanking intronic regions of the* FBN1 *gene, the patient requested a new evaluation as he and his couple were considering the possibility of* in vitro* fertilization with preimplantation genetic diagnosis. Three nonaffected patient's relatives as well as 4752 individuals with phenotypes other than MFS were also genetically tested. Written informed consent was obtained from all subjects before the study. The project was approved by each local ethics committee.

### 2.2. Genomic DNA Extraction

The patient's and relative's genomic DNA was extracted from saliva samples collected with the DNA Sample Collection Kit OG-500 (Oragene), on the QIAsymphony SP robot using the QIAsymphony DNA midi Kit (Qiagen). Purified genomic DNA was quantified using Nanodrop 1000 Spectrophotometer (Thermo Scientific) and DNA integrity was determined on the 2200 TapeStation (Agilent Technologies) following the manufacturer's recommendation.

### 2.3. Next-Generation Sequencing

Custom probe RNA baits (120 nucleotides) for NGS were designed using Agilent's SureDesign online tool to cover all exons and exon/intron boundaries of the human* FBN1* gene. In addition, another 34 genes included in the Health in Code panel (see the Genetic Testing Registry at NCBI, Test ID: GTR000530671.1, www.ncbi.nlm.nih.gov/gtr/tests/530671) related to aortic diseases and phenocopies were NGS analyzed. For NGS library construction, patient genomic DNA (3 *μ*g) was sheared into 150-200 bp fragments by Covaris E220 focused-ultrasonicator. Preparation and capture of NGS libraries were performed on the Bravo instrument (Agilent Technologies) following the manufacturer's instructions. The DNA libraries were normalized to 10 nM and then pooled for multiplexed sequencing (HiSeq 1500, Illumina). Filtering of variants was performed using in-house data sets, the database of single-nucleotide polymorphisms (www.ncbi.nlm.nih.gov/projects/SNP/, build 132), Human Gene Mutation Database (HGMD, http://www.hgmd.cf.ac.uk/ac/index.php), and the Health in Code database. In order to confirm the* FBN1* variants identified by NGS, a standard PCR amplification and bidirectional Sanger sequencing were performed. In silico analysis of identified variants was performed using the splicing prediction module of the Alamut Visual v.2.9.0 software (Interactive Biosoftware), running five independent algorithms for splice signal detection: SpliceSiteFinder-like (SSF), MaxEntScan (MES), NNSPLICE (NNS), GeneSplicer (GSP), and Human Splicing Finder (HSF).

### 2.4. Generation of Allelic Minigene Constructs

*FBN1* allelic minigene fragments (2798 bp), including either the reference (Ref) or mutated (Mut) sequence, have been generated by PCR using the patient genomic DNA as template, Phusion Hot Start II high-fidelity DNA polymerase (Thermo Scientific), and primers 516-519 (Table 1). The agarose-gel purified PCR fragments were cloned into a linearized dual tagged (N-terminal 3xFLAG and C-terminal c-Myc) expression vector (p3xFLAG-Myc-CMV-26, Sigma) as detailed in Supp.  Figure 1A. Plasmids grown in XL1-Blue Supercompetent* E. coli* cells (Stratagene) were purified by using a PureLink HiPure plasmid filter purification kit (Invitrogen) according to the manufacturer's protocol. All the* FBN1* constructs were verified by full-length insert sequencing (for primers, see Table 1). Plasmid 1283 was selected as the* FBN1-*Ref minigene, and plasmid 1288 was selected as the* FBN1*-Mut minigene (Supp.  Figure 1B). Plasmids were formulated at a final DNA concentration of 1 mg/ml in sterile isotonic saline.

### 2.5. Cell Culture and Transfection In Vitro

HeLa (an epithelial cell line derived from human cervical epithelioid carcinoma, passage +4) and COS-7 (a fibroblast-like cell line derived from African green monkey kidney tissue, passage +5) were purchased from the European Collection of Authenticated Cell Cultures (Sigma). Cells were trypsinized at 70–80% confluence, cell numbers were determined using an automated cell counter (Countess, Invitrogen), and 100,000 HeLa or 60,000 COS-7 cells/well were plated in 12-well culture plates, allowed to attach overnight, and transiently transfected with 1.0 *μ*g of plasmid DNA. All transfections were carried out with Lipofectamine 3000 (Invitrogen) following the manufacturer's instructions. For each plasmid, four separate transfection assays were employed, and in each assay transfections were performed in duplicate. The transfection efficiency, evaluated by cotransfection with a CMV-EGFP (enhanced green fluorescent protein) vector followed by fluorescent microscopy, was 70-80%. Equivalent transfection efficiency was verified by cotransfection with a plasmid coding for the N-terminal 3xFLAG-tagged bacterial alkaline phosphatase (BAP) fusion protein (p3XFLAG-CMV-7-BAP, Sigma) followed by Western blot detection with an anti-FLAG antibody. Additional controls included mock and empty vector-transfected cells. In some experiments, the transfected cells were incubated with the cell-permeable proteasome inhibitor MG132 (Sigma) or dimethyl sulfoxide (Sigma) vehicle for 7 hours. The cells were harvested at 24–48 hours after transfection and processed for RNA and protein extraction.

### 2.6. Semiquantitative RT-PCR

Total RNA was extracted by RNeasy-Mini Kit (Qiagen) according to the manufacturer's protocol, subjected to column digestion of DNA with RNase-free DNase (Qiagen), and reverse transcribed using SuperScript IV (Invitrogen) and oligo-dT primers. Semiquantitative RT-PCR was performed in a Biometra II system using the* RPL19* gene (coding for ribosomal L19 protein) for normalization of RT-PCR data [18]. The amount of cDNA and the number of cycles were varied for each primer pair (see Table 1) to ensure amplification within the linear phase. Reactions, including non-RT control and nontemplate control, were performed at least in triplicate. PCR products were visualized on 2% agarose gels by ethidium bromide staining and band intensity was estimated by densitometry (VersaDoc 1000) and Quantity One software (Bio-Rad).

### 2.7. SDS-PAGE and Western Blotting

Cell samples were homogenized and solubilized in standard 2X Laemmli buffer (Invitrogen) supplemented with complete protease inhibitor cocktail (Roche). Following centrifugation at 20,000 g for 30 minutes, the concentration of supernatant proteins was analyzed using the Bio-Rad DC Protein Assay Kit according to the manufacturer's protocol. Extracted proteins were subjected to SDS-PAGE (Mini-Protean-III, Bio-Rad), stained with Coomassie or blotted onto PVDF membranes (Hybond-P, Amersham Bioscience), and probed with mouse monoclonal anti-*Myc* or anti-FLAG antibodies (Sigma). Molecular weight (MW) standards (MARK-12 and SeeBlue Plus2 from Invitrogen) were included on each gel. Equivalence of protein loading was confirmed by Amido Black staining of blots after immunodetection. Blocking, washing, incubation with diluted primary and secondary HRP-conjugated antibodies (Sigma), and visualization of immunodecorated bands by the Super-Signal West Pico PLUS chemiluminescent substrate (Thermo Scientific) were carried out as previously described [19].

## 3. Results

DNA from patient saliva was used for* FBN1* gene targeted NGS analysis with in-house analysis pipeline. Paired end sequencing covered nearly 34,000 bases of* FBN1* encompassing 66 exons along with their flanking (200 bp) intron regions. A total of 8 variants of* FBN1 *were identified with high variant-calling stringency including 7 variants localized in intronic noncoding regions and one synonymous exonic variant which does not influence the amino acid structure of the protein (Table 2). All variants were confirmed by Sanger sequencing. It is worth noting that the identified variants, with the exception of the heterozygous c.2678-15C>A, have been previously described in the database of short genetic variation (dbSNP) and classified as benign or uncertain in the ClinVar database. NGS coverage analysis metrics across the region of interest for* FBN1* allowed for the identification of the heterozygous c.2678-15C>A variant which are shown in Supp.  Figure S2. The c.2678-15C>A variant was not identified by Sanger sequencing in any of the three clinically unaffected patient's relatives. This variant was also not detected in the control population consisting of 4752 individuals with phenotypes other than MFS who were referred to the Health in Code laboratory for NGS analysis. In addition, our NGS screening of a panel of 34 genes related to aortic diseases or involved in the differential diagnosis of MFS did not reveal any pathogenic variant in the case patient.

**Figure 1:**
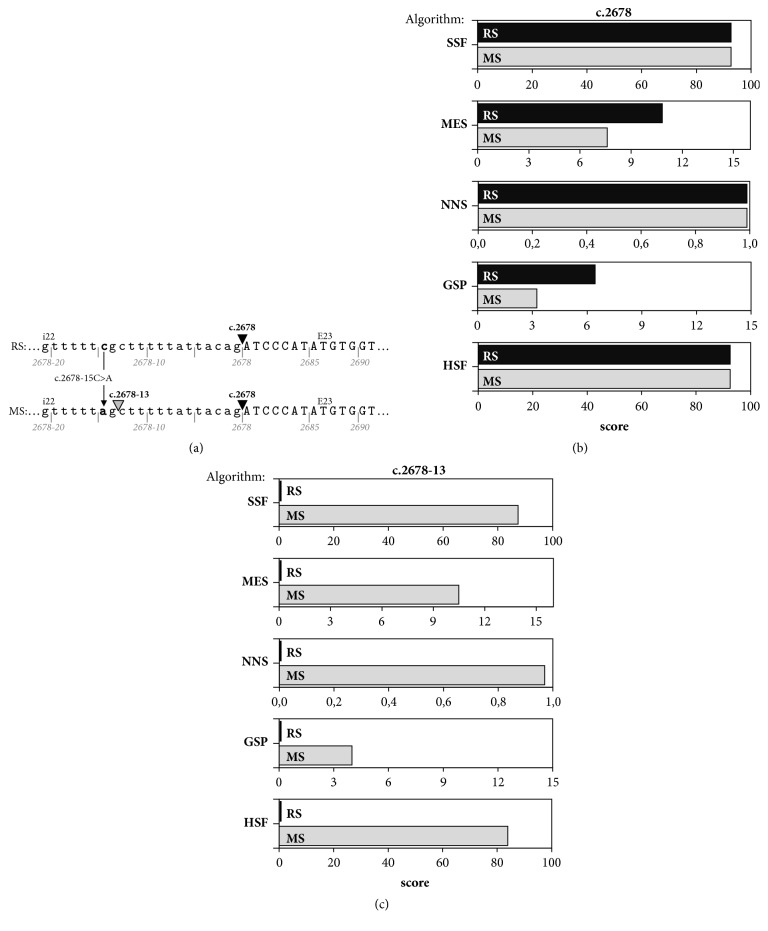
Schematic representation of in silico analysis of the c.2678-15C>A variant identified in intron 22 of the* FBN1* gene by various computational tools. (a) Representation of the position of the canonical (c.2678, black triangle) and cryptic (c.2678-13, grey triangle) splice site identified in the reference (RS) and mutated sequence (MS), respectively. Arrow: the C to A point mutation (c.2678-15C>A). The intron 22 (i22) sequence is shown in lower case and exon 23 (E23) sequence in upper case. (b) The predicted splice score of the canonical site decreases by 29.8% and 49.7% in the mutated sequence (grey parallelogram) versus the reference sequence (black parallelogram) as revealed by the MES and GSP algorithm, respectively. (c) The predicted splice scores of a new cryptic splice site due to the c.2678-15C>A mutation.

To verify the potential role of* FBN1* variants identified by NGS screening, we used the Alamut Visual predictive software. The computational predictions showed that only the heterozygous c.2678-15C>A intronic variant might influence the splicing process by differentially affecting canonical versus cryptic slice site utilization (Figure 1). Relative to the canonical acceptor site c.2678 of the human* FBN1*, MES and GSP programs, respectively, showed a score reduction of approximately 30% and 50% for the c.2678-15C>A intronic variant as compared to reference sequence, while the score values calculated by the remaining three programs (SSF, NNS, and HSF) were estimated to be unaltered (Figure 1(b)). As concerns the cryptic splice site c.2678-13, in contrast, all five Alamut algorithms predicted with high probability (see Figure 1(c)) that the c.2678-15C>A intronic variant would activate a cryptic acceptor splice site within intron 22, at position 13 nucleotides (nt) prior to exon 23.

Taken together, these results strongly suggested that the c.2678-15C>A intronic variant induces a cryptic splice site in intron 22 of the* FBN1* gene that in turn could lead to an inefficient recognition of canonical splice site, giving rise to a frameshift and premature termination codon (PTC) in the* FBN1* mRNA.

**Figure 2:**
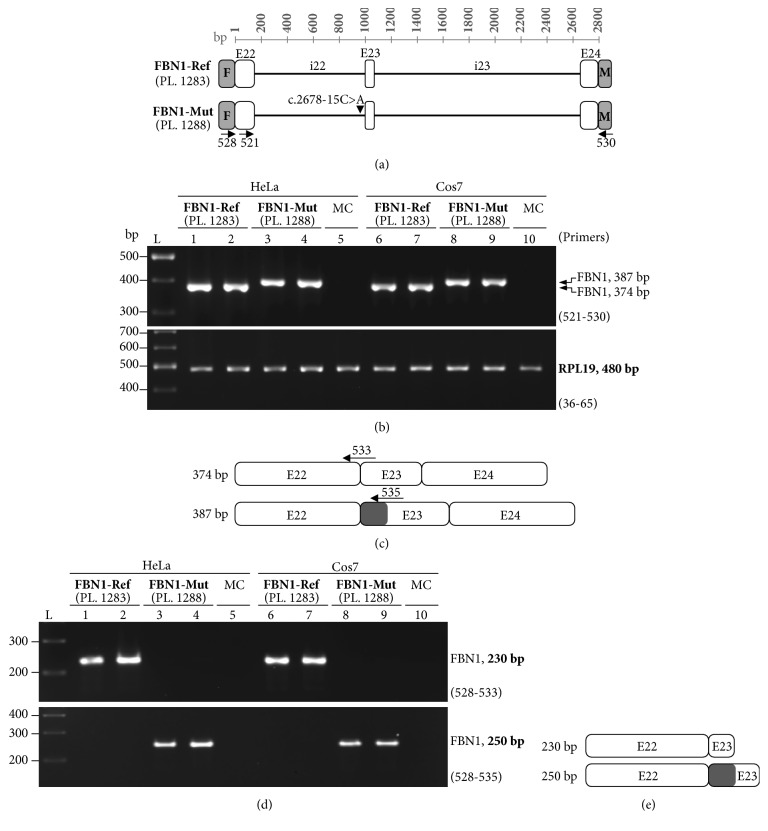
RT-PCR analysis of minigene-derived transcripts. (a) Schematic representation of FLAG (F) and Myc- (M-) tagged reference (*FBN1-*Ref) and mutant (*FBN1*-Mut) minigene plasmids (PL). Exons (E) are denoted with white boxes and introns (i) with solid black horizontal lines. The approximate location of the primers for downstream RT-PCR analysis is shown (for primer sequences see Table 1). (b) Expression of* FBN1-*Ref and* FBN*1-Mut minigenes in HeLa and COS-7 cells as revealed by RT-PCR, using primers (521 and 530) targeting E22 and Myc. A representative of two independent experiments for each transfection is shown.* RPL19* amplification was carried out as an input RNA control for the RT-PCR. L: size reference ladder. MC: mock cells. (c) Schematic representation of PCR products corresponding either to correct splicing (374 bp band) or to partial inclusion (dark grey box) of intron 22 (387 bp band) as revealed by sequencing (see Supp. Figure S3). (d) Partial inclusion of intron 22 in transcripts was assayed by using different combinations of primers targeting FLAG (primer 528 shown in (a)), exon 22/exon 23 junction (primer 533), or exon 23/retained intron 22 inside (primer 535). (e) A diagram of the PCR products (i.e., the 230 bp and 250 bp bands shown in (d)) as revealed by sequence analysis.

To investigate the effect of the* FBN1* splice site variant c.2678-15C>A at the RNA and protein level, we used a minigene approach with reference (Ref) and mutant (Mut) expression plasmids containing the* FBN1* fragments which were amplified from the patient genomic DNA (see Supp.  Figure S1). Each plasmid was transfected into HeLa and COS-7 cells for transient expression. RT-PCR amplifications of the mini-Ref transcript with different primer sets yielded single bands of the expected size (Figure 2(b); 374 bp bands) corresponding to the normal inclusion of exons 22, 23, and 24, with introns 22 and 23 having been removed. The sequencing of these RT-PCR products confirmed that the introns were removed and the exons were correctly ligated together (Figure 2(c) and Supp.  Figure S3A). In contrast, slightly longer 387 bp bands were PCR amplified from HeLa and COS-7 cells transfected with the mini-Mut plasmid (see Figure 2(b)). These RT-PCR products were eluted from the gel and sequenced. The 387 bp mini-Mut band included exon 22, a part (13 nt) of intron 22, and exons 23 and 24 (see Figure 2(c) and Supp.  Figure S3B). The results indicated that the* FBN1* c.2678-15C>A variant affects splicing by promoting the insertion of a 13-nt intron 22-derived sequence in the* FBN1* transcript due to activation of a cryptic splice site localized at c. 2678-13. A more detailed PCR analysis using primers located inside the intron 22-derived insertion (Figure 2(c)) revealed that the aberrantly spliced transcript was expressed in HeLa and COS-7 cells transfected with the mini-Mut plasmid being not detected in cell transfectants expressing mini-Ref plasmids (Figures 2(d) and 2(e)). The latter supports the suggestion that the* FBN1* c.2678-15C>A variant could lead to haploinsufficiency of full-length FBN1 protein.

Overall, the data demonstrated that the c.2678-15C>A variant can repress recognition of the canonical splice site in intron 22 of the* FBN1* gene causing a shift from canonical toward cryptic splicing that can lead to insertion of a 13-nt intron 22-derived sequence, frameshift, and the creation of a PTC (see Supp.  Figure 3B). Following the standards and guidelines for the interpretation of sequence variants [20], the* FBN1* c.2678-15C>A mutation was classified as pathogenic (see Supp.  Table S1) and submitted to the ClinVar database at NCBI (www.ncbi.nlm.nih.gov/clinvar/) with the accession number SCV000611711.

The relative level of transcripts produced in the mini-Ref-containing cells was comparable with that observed in cells transfected with the mini-Mut construct (see Figure 2(b)), suggesting that the aberrantly spliced transcripts may escape, at least partly, from a nonsense-mediated mRNA decay (NMD) under our experimental conditions.

**Figure 3:**
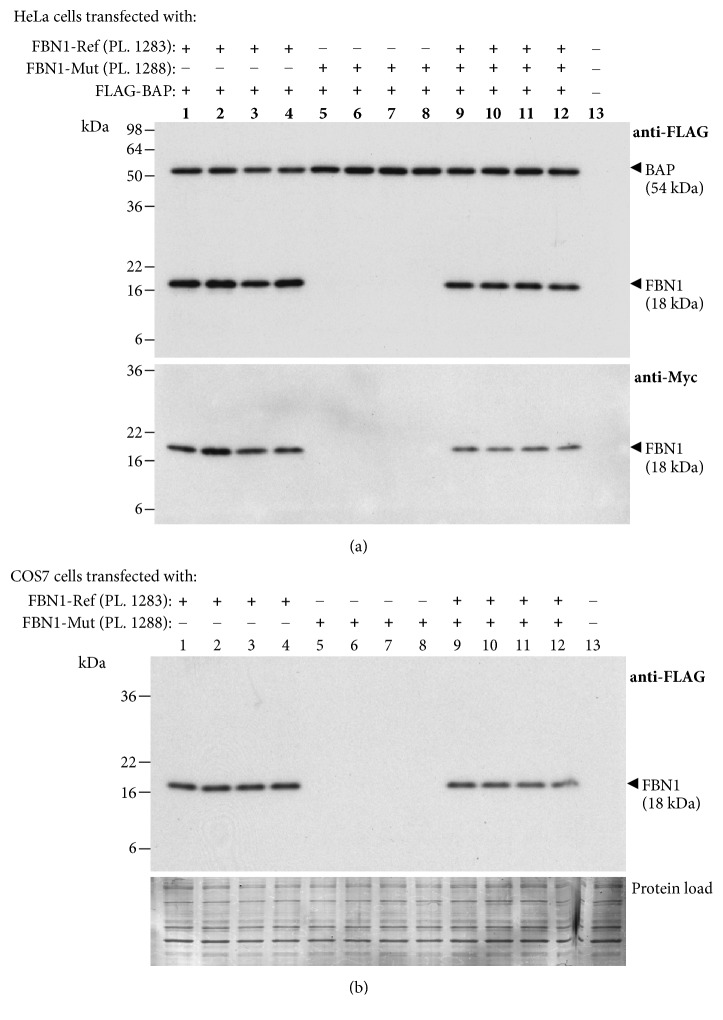
Western blot analysis of minigene-derived proteins. (a) FLAG/Myc-tagged reference (*FBN1-*Ref) and mutant (*FBN1*-Mut) minigene plasmids (PL) were transfected into HeLa cells as indicated at the top of each lane, and cell lysates were analyzed by Western blotting with a mouse monoclonal anti-FLAG (upper panel) or anti-Myc (lower panel) antibody. The results from experiments performed on four batches of cells for each transfection are shown. Lane 13: cells transfected with empty vector. The Western blot detection of the FLAG-tagged bacterial alkaline phosphatase (BAP) was used as a marker of equivalent transfection efficiency and equal loading. MW values (kDa) of the bands detected are shown in brackets. (b) COS-7 cells were transfected as indicated at the top of each lane and analyzed by Western blotting with a mouse monoclonal anti-FLAG antibody (upper panel). Protein load (lower panel): membrane stained with Amido Black 10B after immunostaining. Other indications as in (a).

**Figure 4:**
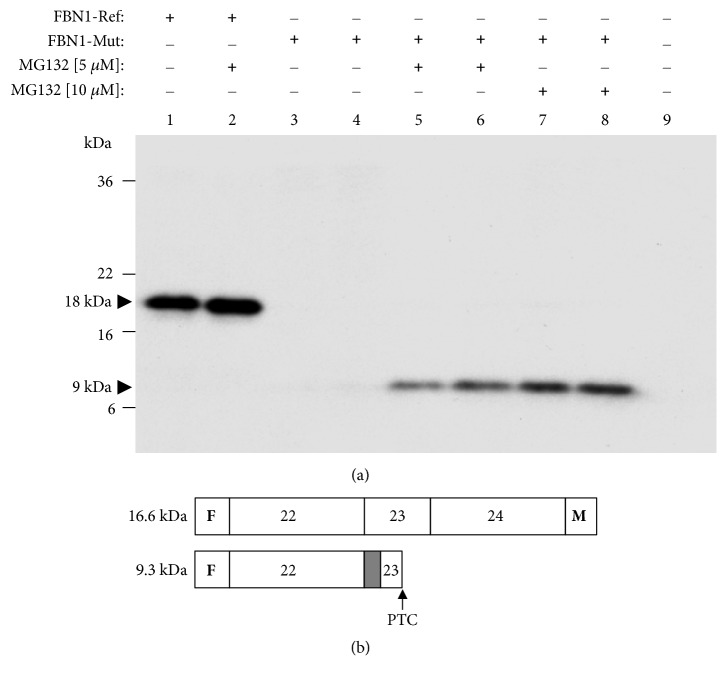
Treatment of transfected cells with MG132 leads to detection of truncated proteins derived from mutant* FBN1* constructs. (a) FLAG/Myc-tagged reference (*FBN1-*Ref; PL 1283) and mutant (*FBN1*-Mut; PL 1288) minigene plasmids (PL) were transfected into HeLa cells as indicated at the top of each lane. Lane 9: mock cells. Either the proteasome inhibitor MG132 or the DMSO vehicle was applied to transfected cells as indicated in Materials and Methods. Whole-cell extracts of transfected cells were analyzed by Western blotting with a mouse monoclonal anti-FLAG antibody. Black arrows: MW values (kDa) of the bands detected. (b) A diagram of the protein products as predicted by sequence analysis of the corresponding cDNAs. Deduced MW values (kDa) are also indicated. Dark grey box: partial inclusion of intron 22; F: FLAG epitope; M: Myc epitope; PTC: premature termination codon.

To verify whether these aberrantly spliced transcripts are translated into protein products, we performed SDS-PAGE and Western blotting of protein lysates of HeLa and COS-7 cells transfected with FLAG/Myc-tagged mini-Ref and mini-Mut plasmids. The 18 kDa bands were detected by both anti-FLAG and anti-Myc antibodies in HeLa (Figure 3(a)) and COS-7 (Figure 3(b)) cells expressing the mini-Ref plasmid 1283. Their apparent molecular weight (MW) values (18 kDa) were found to acceptably match the deduced MW sum of the mini-Ref FBN1 sequence, three FLAG and one c-Myc tags (i.e., 16.6 kDa, see Figure 4(b)). Surprisingly, neither the 18-kDa band nor any others were detectable in cells transfected with the mini-Mut plasmid 1288 (see Figure 3) although these cell transfectants produced high levels of the aberrantly spliced transcript (see Figure 2).

It is widely accepted that aberrant proteins can be rapidly degraded by the ubiquitin proteasome system (UPS). To determine whether inhibition of the UPS may influence the detection of proteins from the aberrantly spliced transcripts, we performed an analysis of proteasome inhibitor MG132 treatment of HeLa cells transfected with mini-Ref and mini-Mut constructs containing three* FBN1* exons with flanking introns. Western blot revealed that MG132 treatment rescues protein expression of the mini-Mut construct in, respectively, transfected cells to levels comparable to those observed in the cells transfected with the mini-Ref plasmid; this rescue effect was dose-dependent (Figure 4). As might be expected in the presence of a PTC in the aberrant transcript (see Supp.  Figure S3B), the corresponding protein band was of a lower MW in comparison with that of the correctly processed mini-Ref construct, i.e., 9 kDa versus 18 kDa, respectively (see Figure 4(a)). Notably, these apparent MW values estimated by SDS-PAGE and Western blotting were very similar to their respective deduced MW values (see Figure 4(b)). Anti-Myc detection did not reveal the expression of the 9 kDa alternative protein in the mutant transfected cells (data not shown) because the* FBN1* c.2678-15C>A variant produced, as predicted, a frame shift and PTC within exon 23. Because of the PTC, the Myc-coding region will not be read-through.

## 4. Discussion

Our study adds to the mutational spectrum of the* FBN1* gene in MFS. Using a previously unrecognized variant in intron 22 of the* FBN1* gene (c.2678-15C>A), revealed by targeted NGS in a MFS patient, we demonstrate, as a proof-of-concept, that this variant causes aberrant splicing, frameshift, and PTC. This pathogenic variant induces aberrant* FBN1* pre-mRNA splicing by the generation of a new cryptic acceptor site that outcompetes the canonical splice site. The single nucleotide C to A change in intron 22 of the* FBN1* is, therefore, considered clinically relevant to MFS. In contrast, the known C to T nucleotide substitution at the same site (i.e., c.2678-15C>T, rs181681840) is not expected to have any clinical significance because its population frequency (1% in the African population; gnomad.broadinstitute.org/variant/15-48786466-G-A) is not consistent with MFS incidence data (approximately 1 case per 5000 individuals [14]), and multiple lines of computational evidence suggest no impact on gene product; the variant c.2678-15C>T is classified as benign in ClinVar (www.ncbi.nlm.nih.gov/clinvar/variation/137303).

Point mutation variants that affect* FBN1* pre-mRNA splicing represent approximately 10% of reported* FBN1* mutations in patients with MFS [12] and are frequently associated with aortic wall alterations [13]. The accumulated data clearly indicate that splice mutations located besides the* FBN1* exon-coding area and canonical exon's splice site motifs also play an important role in MFS [21]. In particular, it was found that patients suffering from MFS can carry* FBN1* intronic variants resulting in cryptic splicing and exonization of intronic sequences at the transcript level [13–16, 22].

In this work, we identified a new intronic variant (c.2678-15C>A) of the* FBN1* gene in a patient with a classic MFS phenotype. The mutation was not identified in his unaffected mother, brother, and paternal aunt and could have been inherited from his deceased father who was retrospectively considered as very likely affected by MFS.

We showed, using minigene functional assays followed by sequencing, that this variant leads to the exonization (insertion) of a part of the intron 22 sequence into* FBN1* transcripts by aberrant splicing. The insertion leads to frameshift and creates a PTC in exon 23 (see Supp.  Figure S3). Mechanistically viewed, this PTC would unlikely be expected to direct the corresponding transcript(s) to NMD, because it is located in a NMD-insensitive region, less than 50 nt upstream of the 3′-most exon-exon junction measured after splicing [23]. Our data strongly suggest that aberrantly spliced transcripts carrying this PTC, located 38 nt upstream of the junction between exons 23 and 24 in the spliced minigene (Supp.  Figure S3B), could escape NMD in transfected cells and express unstable truncated proteins which were only detectable upon proteasome inhibition with MG132.

Although as yet unproved, we believe that the c.2678-15C>A variant would lead to haploinsufficiency of the FBN1 functional protein and structural connective tissue fragility. Our NGS analysis did not reveal any relevant change in a panel of 34 additional genes related to aortic diseases or involved in the differential diagnosis of MFS, supporting the assumption that the c.2678-15C>A variant of the FBN1 can be considered as a main disease-causing variant in the case patient.

## 5. Conclusion

The intronic c.2678-15C>A substitution has not previously been reported, representing therefore a novel* FBN1* gene variant in MFS. Our results demonstrate that the identified intronic variant disrupting normal* FBN1* mRNA splicing is a MFS-associated mutation that should be taken into account in the diagnosis of patients with MFS. On the basis of our results, we suggest that the clinical phenotype associated with the intronic c.2678-15C>A variant is caused by a reduction of wild-type FBN1 protein. Our findings and others highlight the potential importance of intronic* FBN1* variants in causing MFS as well as the continued need for identifying noncoding mutations in the* FBN1* gene.

## Figures and Tables

**Table 1:** Primers used in this study.

**Primer**	**Target**		**F/R**	**Application**	**Sequence (5**′**-3**′**)**	**PCR product**
516	FBN1	e22	F	PCR cloning (Not I)	GTC TGC GGC CGC GAC CAT CAA GGG CAC TTG CTG GC	516-519: 2798 bp
519	FBN1	e24	R	PCR cloning (EcoRV)	CAG CGA TAT CAC AAG ACA GAT CCT TCC TGT GGC ATC	
523	FBN1	i22	F	Sequencing	GTG AAT GCT GGA GGC CAT GAG AT	
524	FBN1	i23	F	Sequencing	CTT CAC AGG GAG AAA TAT GCA GCA GA	
525	FBN1	i23	F	Sequencing	CTC CAT TAG GCA AAC TGG GAA GGA	
480	Vector	5′UTR	F	Sequencing	GCA GAG CTC GTT TAG TGA ACC GTC	
481	Vector	3′UTR	R	Sequencing	GCA ACT TCC AGG GCC AGG AG	
521	FBN1	e22	F	RT-PCR	ACC ATC AAG GGC ACT TGC TGG C	
530	Vector	c-Myc	R	RT-PCR	CCT CAC AGA TCC TCT TCT GAG ATG AGT	521-530: 374 bp
36	RPL19	e2-3	F	RT-PCR	AAC TCC CGT CAG CAG ATC CG	
65	RPL19	e6	R	RT-PCR	CTT GGT CTC TTC CTC CTT GGA	36-65: 480 bp
528	Vector	FLAG	F	RT-PCR	ATG GAC TAC AAA GAC CAT GAC GGT GA	
533	FBN1	e23-22	R	RT-PCR	CCA CAT ATG GGA TCA ACT TGG CAT AG	528-533: 230 bp
535	FBN1	e23-i22	R	RT-PCR	CCT TTA CCA CAT ATG GGA TCT GTA ATA AAA AG	528-535: 250 bp

**Table 2:** Identified *FBN1* variants using targeted NGS.

	**FBN1 variant compared to RS:**		**Location**	**Zygosity**	**SNP ID**	**Frequency** ^**1**^	**Splicing** ^**2**^
	**NC_000015.10**	**NM_000138.4**					
1	g.48610929A>G	c.248-103T>C	intron 3	HZ	rs1018148	0,93234^3^	NA
2	g.48537938C>G	c.539-130G>C	intron 6	HTZ	rs147780575	NR	NA
3	g.48494269G>T	c.2678-15C>A	intron 22	HTZ	NR	NR	A^4^
4	g.48488453G>A	c.3123C>T^5^	exon 26	HTZ	rs576395584	0.00001	NA
5	g.48444487A>T	c.6037+54T>A	intron 49	HZ	rs2303502	0.22331	NA
6	g.48437451T>C	c.6314-64A>G	intron 51	HZ	rs2042746	0.39285	NA
7	g.48428329G>C	c.6997+17C>G	intron 57	HZ	rs363832	0.27742	NA
8	g.48421799G>T	c.7571-113C>A	intron 61	HZ	rs1820488	0,81307^3^	NA

RS: reference sequence; g.: genomic; c.: coding DNA; HZ: homozygous; HTZ: heterozygous; NR: not registered; NA: not affected; A: affected.

^1^Population frequency from gnomAD (Genome Aggregation Database) except ^3^from dbSNP.

^2^Alamut Visual v.2.9 predictions.

^4^Activation of a cryptic acceptor splice site at c.2678-13 (see Figure 3).

^5^NP_000129.3:p.(His1041=).

## Data Availability

All data are presented in the manuscript.

## Supplementary Materials

Supplementary Materials Supp. Table S1: classifying the* FBN1 *c.2678-15C>A variant as pathogenic. Supp. Figure S1: vector mapping and sequencing analysis of plasmids. Supp. Figure S2: NGS identification of the intronic* FBN1* c.2678-15C>A variant. Supp. Figure S3: direct sequencing analysis of RT-PCR products amplified with primer sets targeting the minigene transcripts in the transfected HeLa cells.
