# Supplementary material for: A Novel Heterozygous Intronic Mutation in the FBN1 Gene Contributes to FBN1 RNA Missplicing Events in the Marfan Syndrome
Source: Biomed Res Int. 2018 May 29;2018:3536495. doi: 10.1155/2018/3536495 (PMC5996431; doi:10.1155/2018/3536495)
Supplement: Supplementary Materials — Supp. Table S1: classifying the FBN1 c.2678-15C>A variant as pathogenic. Supp. Figure S1: vector mapping and sequencing analysis of plasmids. Supp. Figure S2: NGS identification of the intronic FBN1 c.2678-15C>A variant. Supp. Figure S3: direct sequencing analysis of RT-PCR products amplified with primer sets targeting the minigene transcripts in the transfected HeLa cells. [file 3536495.f1.pdf]

Supp. Table S1. Classifying the *FBN1* c.2678-15C>A variant as pathogenic

Type of evidence	Criteria	Evidence of pathogenicity	Evidence code
Population data	Absent in population databases	moderate	PM2
Predictive data	Predicted null variant in a gene where LOF is a known mechanism of disease	very strong	PVS1
Predictive data	Computational evidence support a deleterios effect on the gene product	supporting	PP3
Functional data	*In vitro* functional studies supportive of a damaging effect on the gene product	strong	PS3
Other data	Patient's phenotype is highly specific for a disease with a single genetic etiology	supporting	PP4

LOF - loss of function; PM - pathogenic moderate; PVS - pathogenic very strong; PP - pathogenic supporting; PS - pathogenic strong. A variant is considered pathogenic if the following criteria are met: 1 PVS1 + ≥1 Strong (PS1–PS4) or 1 PVS1 + 1 Moderate (PM1–PM6) and 1 supporting (PP1–PP5) or 1 PVS1 + ≥2 Supporting (PP1–PP5), according to Richards et al., 2015 [20].

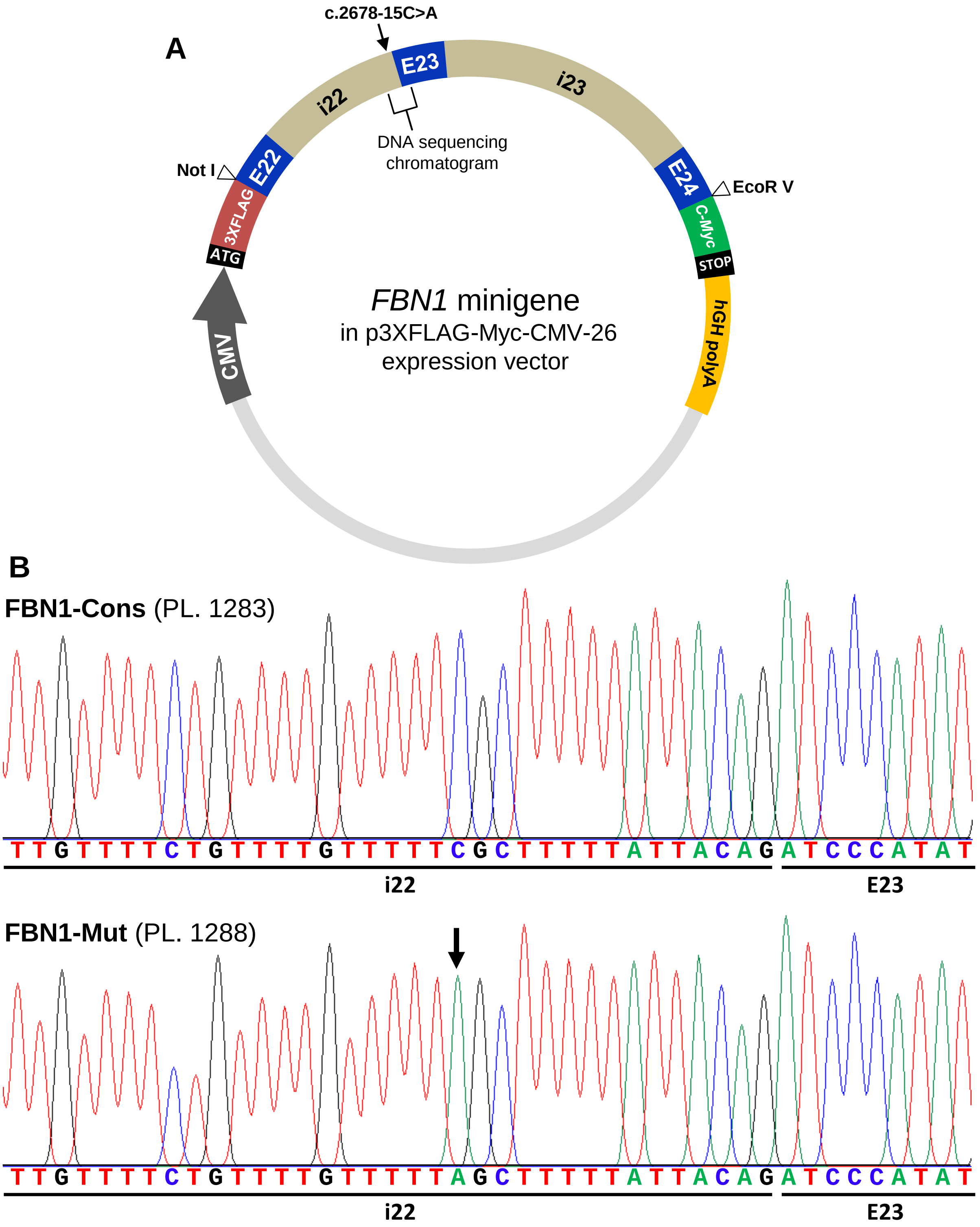
A
c.2678-15C>A
E23
i22
i23
DNA sequencing chromatogram
Not I
E22
3XFLAG
ATG
CMV
E24
EcoR V
C-Myc
STOP
hGH polyA
FBN1 minigene
in p3XFLAG-Myc-CMV-26
expression vector
B
FBN1-Cons (PL. 1283)
TTGTTTTCTGTTTTGTTTTTCGCTTTTTATTACAGATCCCATAT
i22
E23
FBN1-Mut (PL. 1288)
TTGTTTTCTGTTTTGTTTTTAGCTTTTTATTACAGATCCCATAT
i22
E23

Fig. S1

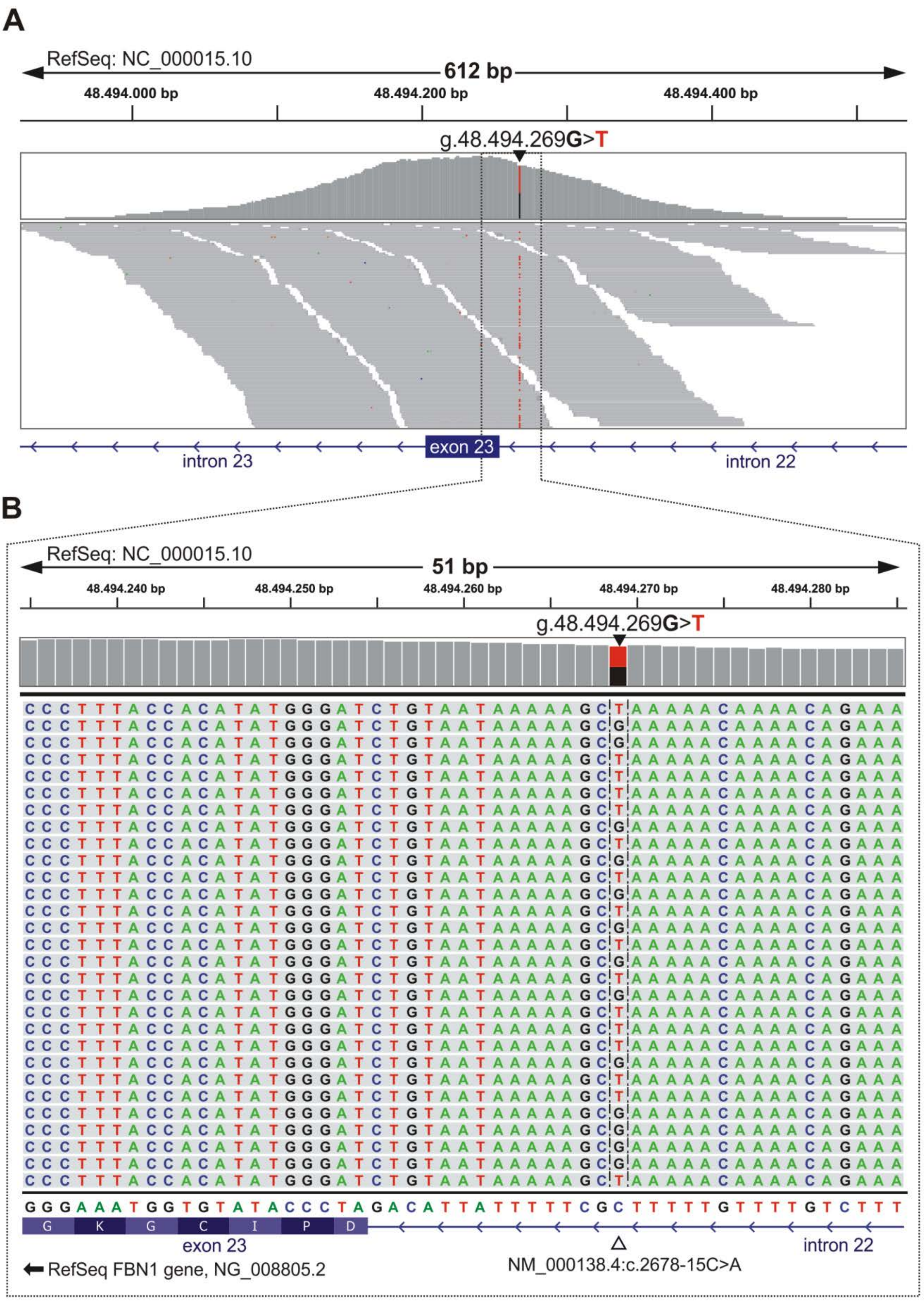

A
RefSeq: NC_000015.10
612 bp
48.494.000 bp
48.494.200 bp
48.494.400 bp
g.48.494.269G>T
exon 23
intron 23
intron 22
B
RefSeq: NC_000015.10
51 bp
48.494.240 bp
48.494.250 bp
48.494.260 bp
48.494.270 bp
48.494.280 bp
g.48.494.269G>T
GGGAAATGGTGTATACCCTAGACATTATTTTTCGCTTTTTGTTTTGTCTTT
G K G C I P D
exon 23
intron 22
RefSeq FBN1 gene, NG_008805.2
NM_000138.4:c.2678-15C>A

Fig. S2

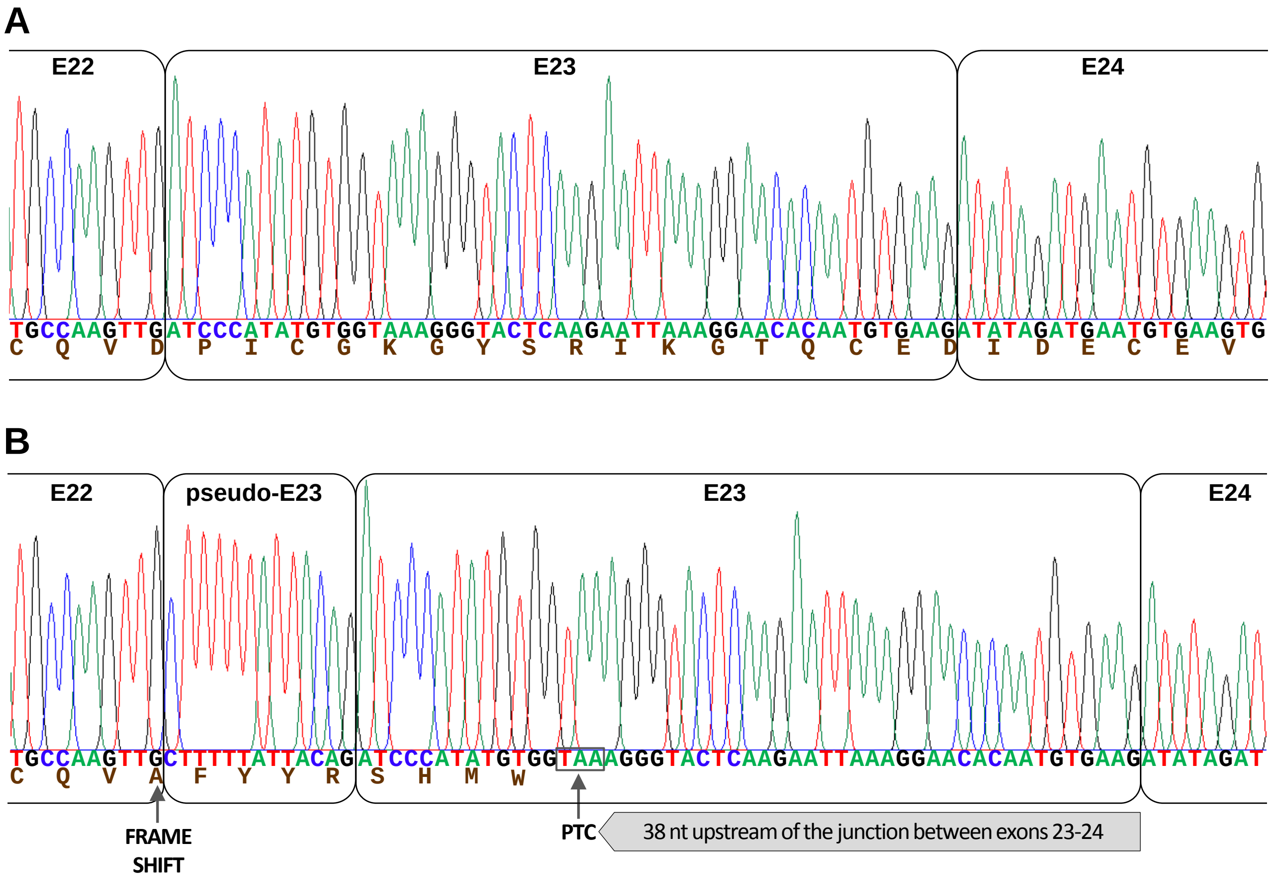
A
E22
E23
E24
TGCCAAGTTGATCCCATATGTGGTAAAGGGTACTCAAGAATTAAAGGAACACAATGTGAAGATATAGATGAATGTGAAGTG
C Q V D P I C G K G Y S R I K G T Q C E D I D E C E V
B
E22
pseudo-E23
E23
E24
TGCCAAGTTGCTTTTTATTACAGATCCCATATGTGGTAAAGGGTACTCAAGAATTAAAGGAACACAATGTGAAGATATAGAT
C Q V A F Y Y R S H M W
FRAME SHIFT
PTC
38 nt upstream of the junction between exons 23-24

Fig. S3
